# Metformin use and the risk of total knee replacement among diabetic patients: a propensity-score-matched retrospective cohort study

**DOI:** 10.1038/s41598-022-15871-7

**Published:** 2022-07-07

**Authors:** Francisco T. T. Lai, Benjamin H. K. Yip, David J. Hunter, David P. Rabago, Christian D. Mallen, Eng-Kiong Yeoh, Samuel Y. S. Wong, Regina WS. Sit

**Affiliations:** 1 The Jockey Club School of Public Health and Primary Care, Faculty of Medicine, Postgraduate Education Centre, Prince of Wales Hospital, The Chinese University of Hong Kong, 30-32 Ngan Shing Street, Sha Tin, New Territories, Hong Kong China; 2 Department of Pharmacology and Pharmacy, Centre for Safe Medication Practice and Research, Li Ka Shing Faculty of Medicine, The University of Hong Kong, Hong Kong, China; 3 Institute of Bone and Joint Research, Faculty of Medicine and Health, The University of Sydney, Sydney, NSW Australia; 4 Department of Family and Community Medicine, Penn State University, Hershey, PA USA; 5 School of Medicine, Keele University, Keele, UK

**Keywords:** Therapeutics, Medical research, Rheumatology

## Abstract

Metformin has been shown to modulate meta-inflammation, an important pathogenesis in knee osteoarthritis (OA). The study aimed to test the association between regular metformin use with total knee replacement (TKR) in patients with diabetes. This is a retrospective study with electronic records retrieved in Hong Kong public primary care. Patients with diabetes aged ≥ 45 who visited during 2007 to 2010, were followed up for a four-year period from 2011 to 2014 to determine the incidence of TKR. Propensity score matching based on age, sex, co-medications and chronic conditions was conducted to adjust for confounding. Cox regression was implemented to examine the association between metformin use and TKR. In total, 196,930 patients were eligible and 93,330 regular metformin users (defined as ≥ 4 prescriptions over the previous year) and non-users were matched. Among 46,665 regular users, 184 TKRs were conducted, 17.1% fewer than that among non-users. Cox regression showed that regular metformin users had a 19%-lower hazard of TKR [hazard ratio (HR) = 0.81, 95% confidence interval: 0.67 to 0.98, P = 0.033], with a dose–response relationship. Findings suggest a potential protective effect of metformin on knee OA progression and later TKR incidence among diabetic patients.

## Introduction

Knee osteoarthritis (OA) is the most common form of chronic arthritis and a leading cause of pain and disability worldwide. According to the Global Burden Disease, the global age-standardized point prevalence and annual incidence rate of OA in 2017 were 3754.2 and 181.2 per 100,000, respectively^1^. Individuals with knee OA have greater pain, activity limitations, psychological distress and markedly reduced quality of life^2,3^. The global age-standardized years lost due to disability rate in 2017 being 118.8, an increase of 9.6% from 1990^1^. Total knee replacement (TKR) is effective, but is costly and carries operation risks; therefore, it is mostly reserved for end-stage knee OA^4,5^. In 2018, the United States Food and Drug Administration recognized OA as a serious disease with an unmet medical need for therapies that modify the underlying pathophysiology and potentially change its natural course to prevent long-term disability^6^. Hence, the search for safe and effective therapeutic options for knee OA remains a top priority in clinical practice and research.

Metformin is a safe, well-tolerated oral biguanide widely used internationally as first-line therapy for type 2 diabetes for over 50 years. In addition to its glucose-lowering effects in type 2 diabetes mellitus (DM), metformin also modulates inflammatory and metabolic factors, resulting in reduced inflammation and plasma lipids levels^7^. Its role in weight reduction has been demonstrated in both diabetics and non-diabetic populations^8–11^. Metformin, a well-known adenosine monophosphate-activated kinase (AMPK) activator, can suppress cyclooxygenase-2 (COX-2) and inducible nitric oxide synthase (iNOS) mRNA and protein expression in a dose-dependent pathway^12^. Its mechanism on the reduction of pain intensity in many inflammatory disorders is explained by its inhibitory effects on the level of pro-inflammatory mediators, thus reducing the level of inflammatory cytokines including TNF-α, IL-1β, IL-6, IL-10 and adipokines^13^. The suppressed level of COX-2 and iNOS also reduces the levels of NO and PGE2 in cell culture media^14^. The above anti-inflammatory and anti-oxidative effect of metformin on synovial joint tissue may reduce pain based on the metabolic regulation of inflammation in OA^15^.

The therapeutic use of metformin in type 2 diabetic patients with OA has been advocated recently, given that they share similar pathogenic risk factors such as aging, obesity, and cytokine- and adipokine-mediated inflammation^16^. Based on the biological effects of metformin on weight reduction and meta-inflammation, regular metformin use may potentially slow down the progression of knee OA. Therefore, we used the receipt of TKR as an endpoint and as a surrogate for OA progression, and conducted a propensity score matched retrospective cohort study to evaluate the effect of metformin on the incidence of TKR using secondary data from a government electronic clinical database.

## Results

### Descriptive results

**Figure 1:**
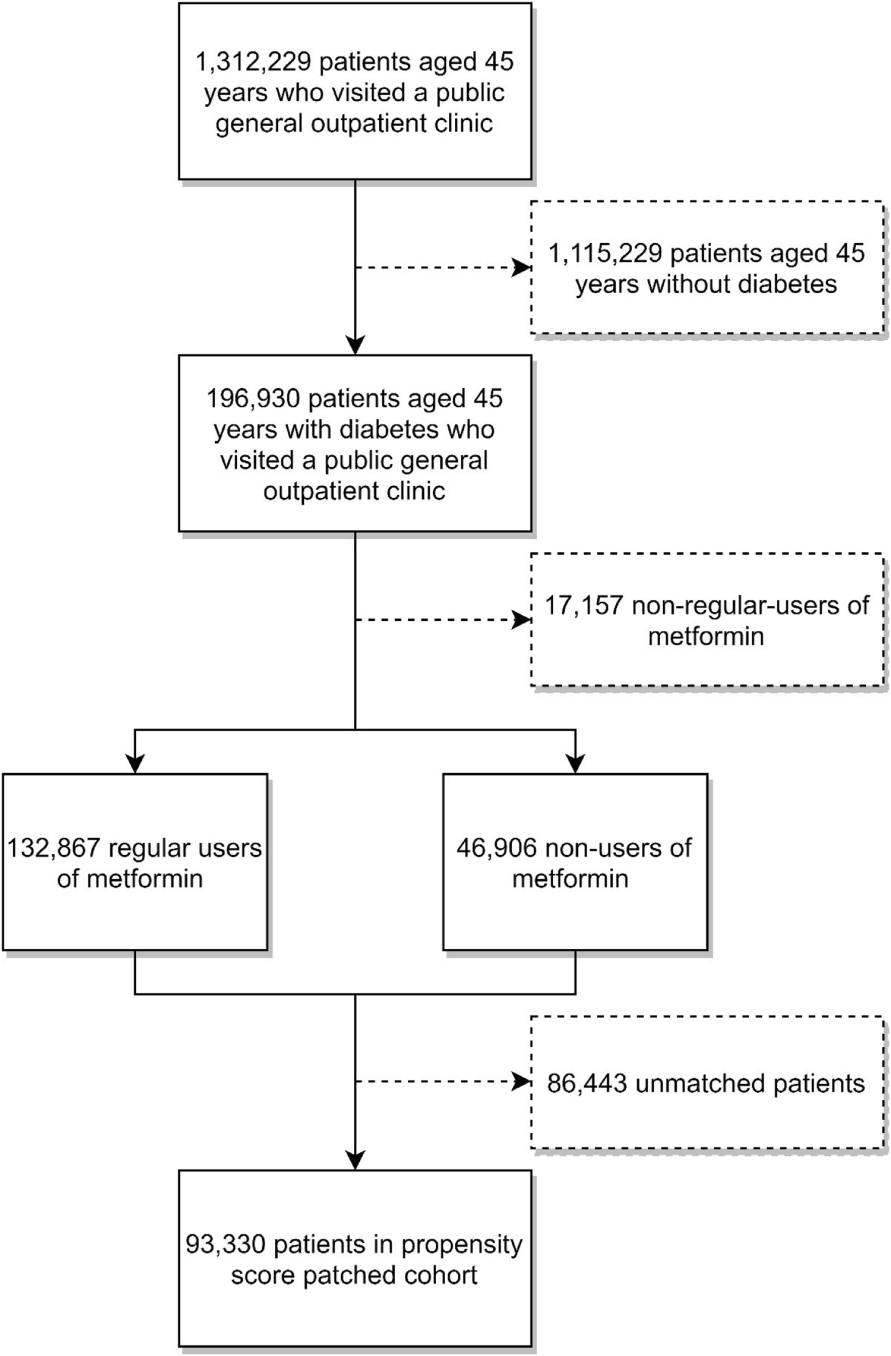
Sample selection procedures to form the final cohort.

From January 1st, 2007 to December 31st, 2010, a total of 15,054,785 general outpatient clinic visits by 1,312,229 patients aged 45 or above were recorded. We removed 1,115,299 who did not have diabetes. The remaining 196,930 diabetic patients included 132,867 regular users and 46,906 non-users of metformin, both of which were included for propensity score matching. A matched cohort of 93,330 patients was used as the final cohort, with 46,665 regular metformin users and 46,665 non-users. The mean (standard deviation, SD) follow-up period was 1,331.6 (362.3) days. Figure 1 shows the procedures of cohort selection for this study.

**Figure 2:**
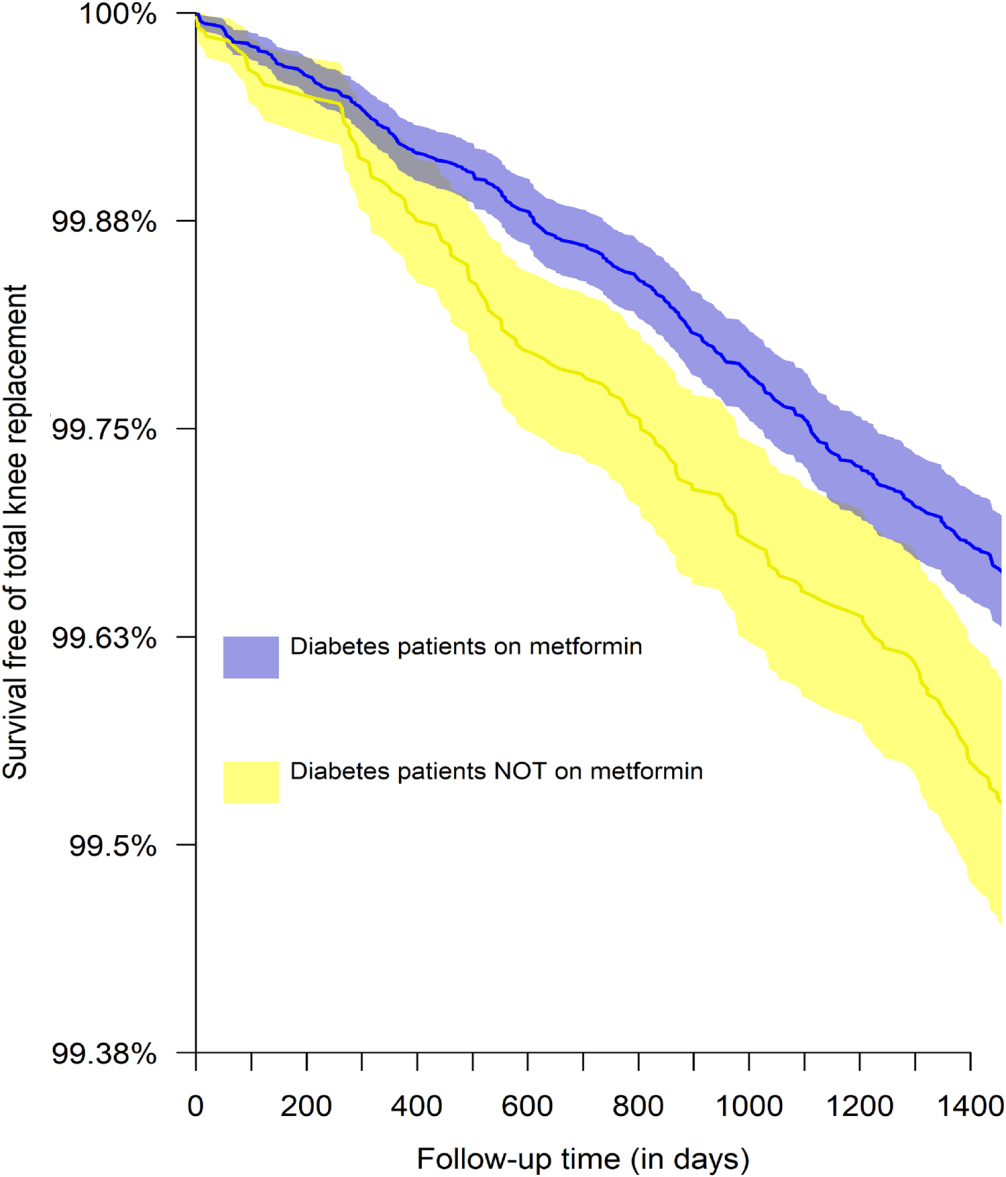
Kaplan–Meier curve illustrating the pattern of survival free of total knee replacement by metformin using status.

Table 1 shows the descriptive statistics of the non-matched and propensity score matched cohorts stratified by metformin use status. After propensity score matching, the standardized (or raw) differences of all potential confounders were less than 0.1, which was indicative of a balance match. Among regular users of metformin, 184 of these patients were admitted for TKR within four years, 17.1% fewer than that among non-users, which had 222 TKRs. Figure 2 shows the Kaplan–Meier curve for total knee replacement among regular users and non-users of metformin.

**Table 1:** Baseline characteristics of propensity score-matched cohort and non-matched cohort.

Metformin use	Non-matched cohort		Matched cohort		
	Non-user	Regular user	Non-user	Regular user	Standardized mean difference
n	46,906	132,867	46,665	46,665	
**Sex (%)**					0.007
Men	21,203 (45.2)	60,906 (45.8)	21,126 (45.3)	20,956 (44.9)	
Women	25,703 (54.8)	71,961 (54.2)	25,539 (54.7)	25,709 (55.1)	
Mean age (standard deviation)	70.1 (11.8)	66.9 (10.9)	70 (11.7)	70 (11.5)	0.002
**Medications (%)**					
Insulin	1416 (3)	4027 (3)	1405 (3)	1313 (2.8)	0.012
NSAIDs	6332 (13.5)	17,561 (13.2)	6308 (13.5)	6128 (13.1)	0.011
Sulfonylureas	20,045 (42.7)	75,912 (57.1)	20,029 (42.9)	19,462 (41.7)	0.025
Paracetamol	16,399 (35)	44,243 (33.3)	16,304 (34.9)	16,075 (34.4)	0.010
**Chronic conditions (%)**					
Renal failure	749 (1.6)	1285 (1)	730 (1.6)	662 (1.4)	0.012
Heart failure	413 (0.9)	432 (0.3)	355 (0.8)	322 (0.7)	0.008
Hypertension	34,459 (73.5)	95,015 (71.5)	34,271 (73.4)	34,516 (74)	0.012
Stroke	1784 (3.8)	2983 (2.2)	1724 (3.7)	1605 (3.4)	0.014
Ischemic heart disease	1711 (3.6)	3329 (2.5)	1662 (3.6)	1574 (3.4)	0.010
Tobacco abuse	290 (0.6)	1421 (1.1)	290 (0.6)	268 (0.6)	0.006
Lipid disorder	9542 (20.3)	29,059 (21.9)	9527 (20.4)	9392 (20.1)	0.007
Total knee replacement (%)	222 (0.5)	505 (0.4)	222 (0.5)	184 (0.4)	
Hazard ratio of total knee replacement (95% confidence interval)	**0.81 (0.67–0.98), P = 0.033**

For the sensitivity analyses with blood pressure and HbA1c included for propensity score matching, number of TKR cases was 4 (0.1%) among regular metformin users and 20 (0.6%) among non-users (Supplementary Table S1), and 17 (0.3%) among regular sulphonylureas users and 20 (0.3%) among non-users (Supplementary Table S2).

### Cox regression results

Results of the Cox regression suggested that the hazard ratio (HR) of TKR for regular metformin users compared with non-users was estimated at 0.81 [95% confidence interval (CI) 0.67 to 0.98, P = 0.033] (Table 1). Sub-analysis with HbA1c and blood pressure included for matching showed the hazard ratio was estimated at 0.20 (95% CI 0.07 to 0.58) (Supplementary Table 1). The association between regular use of sulphonylureas and TKR was non-significant (HR = 1.50, 95% CI 0.79 to 2.85, P = 0.216. (Supplementary Table 2).

As shown in Table 2, compared with zero prescription of metformin, regular metformin use operationalized as 2 + prescriptions in the past year (n = 93,446) had an HR of 0.89 (95% CI 0.74 to 1.08), 3 + prescriptions (n = 93,374) had an HR of 0.85 (95% CI 0.70 to 1.03), and 5 + prescriptions (n = 93,218) had an HR of 0.74 (95% CI 0.61 to 0.91).

**Table 2:** Number of metformin prescriptions and hazard ratio on total knee replacement.

Operationalization of regular metformin use	N (matched cohort) *	Number of total knee replacements among regular metformin users (total number in cohort)	Hazard ratio (95% confidence interval)
Two prescriptions or more in past year	93,446	203 (425)	0.89 (0.74–1.08)
Three prescriptions or more in past year	93,374	193 (415)	0.85 (0.70–1.03)
Four prescriptions or more in past year ^†^	93,330	184 (406)	0.81 (0.67–0.98)
Five precriptions or more in past year	93,218	168 (390)	0.74 (0.61–0.91)

*All cohorts were one-to-one matched cohorts.

^†^Main analysis.

## Discussion

Despite the low incidence of TKR and a relatively short four years follow up period, we found a statistically significant 19%-reduction in the rate of TKR in a diabetic population who are regular metformin users compared to non-users, with an apparent dose–response relationship. Sensitivity analysis with blood pressure and hemoglobin A1c readings included for propensity score matching also reported significant results; no risk reduction was identified for sulphonylureas.

Our results were consistent with another retrospective cohort study among a Taiwanese population with a sample size of 968 in 2018. Patients with OA and DM taking a combination of COX-2 inhibitors and metformin had a 26% reduced risk of joint replacement compared with COX-2 inhibitors alone over 10 years^17^. In another prospective cohort study in 2019, which included 818 participants with obesity and knee OA under the Osteoarthritis Initiatives, the use of metformin was associated with a statistically significant reduced rate of medial knee cartilage volume loss over 4 years and a statistically non-significant trend towards reduced risk of TKR over 6 years^18^. The effectiveness of metformin in knee OA has been tested in a randomized controlled trial at Iraq in 2014; the study found a combination of metformin and meloxicam resulted in greater improvement in knee pain and function compared with meloxicam alone in patients with knee OA at 12 weeks^19^. However, the trial was limited by the small sample size, high dropout rate, short study period, and an unclear data stewardship process.

In knee OA, increased loading in response to mechanical stress has been shown to inhibit cartilage matrix synthesis and induce the expression of pro-inflammatory factors (e.g. cyclooxygenase 2, nitric oxide [NO], IL-1β and prostaglandin E_2_ [PGE_2_])^20,21^ and degradative enzymes (e.g. matrix metalloproteinase (MMP)3, MMP13 and the aggrecanase ADAMTS-5) in chondrocytes^22,23^, leading to cartilage matrix impairment and subchondral bone remodeling^24^. Furthermore, adipose tissue in overweight and obese patients has been proven to release a number of adipokines, such as leptin, adiponectin, visfatin and resistin. The high level of adipokines in serum and synovial fluid, and the subsequent release of pro-inflammatory factors (e.g. NO, IL-6, IL-8, insulin-like growth factor 1, transforming growth factor β) and degenerative enzymes (e.g.MMP9–13), has been shown to destroy chondrocytes, synovial fibroblasts and osteoblasts^24,25^. Metformin, with its potential role in weight reduction, regulation of meta-inflammation, and an effective diabetic control, may possibly delay the progression of knee OA, thus the subsequent need of TKR^26^.

Strengths of the current study include the use of propensity score matching to minimize differences between the two groups. We also included all the confounding factors which might otherwise affected the prescription of metformin, such as diabetic control and comorbid medical conditions such as renal failure and heart failure. The knee pain severity was an important factor that would otherwise affect the outcome of TKR, and it was controlled indirectly by balancing prescriptions such as NSAIDs and paracetamol.

There are several limitations of this study. First, there was limited information on the patient exposure to metformin, including duration of use, dosage and compliance; besides, we were not able to know why some patients were not prescribed metformin, Second, knee OA severity as measured by imaging or pain scores were not available in the retrieved electronic records, which would be an important factor on the outcome of TKR. Third, lifestyle factors and sociodemographic information, which might be related to TKR incidence, was not available in the dataset to investigate any intermediary mechanisms of the association. Likewise, changes in body weight was unavailable to confirm the speculation of metformin’s effect through weight loss. With this number of TKR, we were unable to examine the interaction between metformin use and other co-medications and conduct further sub-group analyses. Fourth, private healthcare sector data were not accessible, and we might have omitted TKR conducted in private facilities. Fifth, we did not use coding of knee OA to define the baseline population because the disease was generally undercoded^27^; instead, we used DM as it is commonly and correctly coded in local clinical practice^28^, and allowed us to include a larger population at risk of having TKR. Sixth, only confounders available in the database can be matched; we cannot adjust or controlled for unmeasured confounders which can only be done by a randomized controlled trial. Finally, we did not know whether all TKRs were for patients with knee OA; nevertheless, local study has shown that the 95% of the TKR in public hospitals were for patients with primary knee OA^29^.

Future research should include longitudinal studies of longer follow up period, more specific selection of patients with preexisting diagnoses of knee OA and its severity, and include dosage information of metformin. Since we did not know clearly the reasons why patients with diabetes were not put on metformin, collection of other baseline characteristics, such as baseline creatinine clearance rate, individual disease profiles and tracking of body weight might be useful. Our findings of potential beneficial effects of metformin on knee OA progression informs the design of a planned phase II randomized controlled trial, which can evaluate the use of metformin in non-DM patients with knee OA, especially among those with comorbid obesity. Metformin is widely used, easily accessible, relatively inexpensive, and has an excellent safety profile. Its use in clinical practice, apart from DM, has been demonstrated in the prevention of type II DM in high-risk adults, reduction of body weight in non-diabetic obese populations, prevention of weight gain associated with the use of antipsychotics in pediatric and adult populations, and in treatment of non-alcoholic fatty liver disease^10,30,31^. The use of metformin has also been repurposed in cancer prevention^32,33^. The antineoplastic effects of metformin have been documented by mechanistic studies^34^ and by ongoing clinical trials^35^. Metformin has been shown to have ancillary effects which are as clinically relevant as the well-known anti-hyperglycemic action. Several reviews have demonstrated evidence in favor of metformin as an endothelial protector^36^, as an effective drug in heart failure^37^, as an anti-inflammatory useful in rheumatological/immunological diseases^38^, and in general as a beneficial medication against numerous aging-related morbidities^39^. Therefore, it is expected that metformin will have a high level of acceptance and uptake in obese non-DM adults with knee OA.

## Methods

This study was approved by the Survey and Behavioral Ethics Committee of the Chinese University of Hong Kong (Project Code: Elderly Care—CUHK) and we confirm that all research was performed in accordance with relevant guidelines/regulations. As only secondary analysis of the anonymized patient records was involved, no written consent was required as approved by the aforementioned Committee.

### Study design and data source

A retrospective cohort design was used in this study, which was extended from a broader project on health care services commissioned by the Government of the Hong Kong Special Administrative Region. The data were extracted from an electronic clinical database, the Clinical Data Analysis and Reporting System (CDARS) of the Hospital Authority (HA), which contains health care data gathered prospectively in the public care setting since 1999. The HA covered 6 million people in the public primary care settings in 2018–2019, and provides up to 80–90% of all secondary and tertiary care in Hong Kong and should therefore be representative of the general population of Hong Kong^40^. This computerized system is the only portal of information entry in all public health care settings across all geographical regions of Hong Kong (i.e. the New Territories, Kowloon and Hong Kong Island). In all clinical consultations, medical doctors entered prescription details as part of their routine practice. The details were subsequently sent to pharmacy professionals for drug dispensing. This electronic patient record system captured all amendments of prescriptions following the attending physicians' consultations.

We were granted access to the routine electronic health records of patients aged 45 or older from the public sector. A closed cohort was formed with data extractions from records of all patients with type 2 diabetes (International Classification of Primary Care (ICPC) codes T90) aged 45 or more who visited any of the 74 public primary care clinics run by the HA during 1st January 2007 to 31st December 2010. The last visit of these patients during the period was used as the index date (baseline) and we retrieved the corresponding outpatient clinical records over one year prior to the index date. Patients were then followed up until admission to any public hospitals for TKR replacement surgeries [defined using International Classification of Diseases, Ninth Revision (ICD-9) 81.54], in-hospital deaths, or four years after the baseline, starting from 1st January 2011 to 31st December 2014.

### Main exposure (regular metformin use)

Regular metformin users were defined as patients who had ≥ 4 prescriptions over one year prior to the baseline. In local public primary care, patients with chronic illnesses were followed up every 3 to 4 months. Therefore, patients who have had ≥ 4 prescriptions of metformin would imply regular use of at least one year. Patients who were not prescribed metformin in the past year were identified as non-users.

### Propensity score matching

One-to-one propensity score matching (regular users versus non-users) was conducted using the nearest-neighbor approach based on a range of potential confounding factors (caliper < 0.1). Specifically, we first included baseline comorbidities according to records over one year prior to the baseline: hypertension (ICPC codes K86 and K87), ischemic heart disease (ICPC codes K74, K75, and K76), stroke (ICPC codes: K89 and K90), renal failure (ICPC code U88 and U99), chronic heart failure (ICPC code K77), lipid disorder (ICPC code T93) and tobacco abuse (ICPC code P17). Second, co-medications in the past year included any prescription of sulphonylureas (gliclazide, glimepiride, or glipizide), insulin (including human insulin, insulin isophane human, insulin neutral human), paracetamol, and nonsteroidal anti-inflammatory drugs (diclofenac, ibuprofen, indomethacin, naproxen, or mefenamic acid) were considered. Hypoglycemic agents such as sodium-glucose cotransporter-2 (SGLT2) inhibitors or glucagon-like peptide 1 (GLP-1) analogues were not included as they were available in the GOPC drug formulary during the study period. Opioid drugs were not included because they were not prescribed in local primary care. Third, age and sex were also included. We checked the balance of the propensity score matched cohort using the standardized mean differences (or raw differences for proportions) of these variables with a difference less than 0.1 indicating balance^41^. R package ‘MatchIt’ was used to implement the propensity score matching algorithms^42^.

### Study outcome (TKR)

The study outcome was the incidence of the first TKR (ICD-9 CM: 81.54) conducted in public hospitals during the study period from 1st January 2011 to 31st December 2014. Revisions of TKR (ICD-9 CM: 00.80) was not counted as an outcome.

### Statistical analysis

We ran a Cox proportional hazard regression to estimate the hazard ratio of TKR for regular metformin users compared to non-users using the propensity score matching. Potential confounders with post-matching standardized mean difference > 0.1 were included in the regression as covariates. As a sensitivity analysis, we conducted a supplemental analysis on a sub-sample with systolic and diastolic blood pressure readings and hemoglobin A1c (HbA1c) readings for propensity score matching. As only a fraction of the patients had this information, it was not included in the main analysis. In addition, we also replicated this sub-analysis on the regular use of sulphonylureas, which was defined as ≥ 4 prescriptions over the past year (versus non-users with zero prescriptions) and examined its association with TKR. Finally, the main analysis was replicated with regular metformin use reoperationalized as 2 + , 3 + , and 5 + prescriptions in the past year to examine the potential dose–response relationship in the association with four new propensity score matched cohorts. All analyses were conducted using R, version 3.6.0 (R Foundation for Statistical Computing, Vienna, Austria)^43^. There were no missing data on age and sex, and patient records without any codes of diagnoses or prescription records were treated as absence of chronic conditions or medications.

### Ethics approval and consent to participate

This study was approved by the Survey and Behavioral Ethics Committee of the Chinese University of Hong Kong (Project Code: Elderly Care—CUHK). As only secondary analysis of the anonymized patient records was involved, no written consent was required.

## Conclusion

Our findings suggest a potential slowing of OA disease progression among type 2 diabetic patients associated with the regular use of metformin as assessed by the incidence of TKR. With the proposed biological effects on meta-inflammation and weight reduction, metformin may be repurposed as a disease-modifying agent for patients with knee OA. Further study is warranted to further elucidate biological mechanism and a potential clinical role.

## Supplementary Information

Supplementary Information.

## Data Availability

The datasets used and analysed during the current study are available from the corresponding author on reasonable request.
