# Supplementary material for: Metformin use and the risk of total knee replacement among diabetic patients: a propensity-score-matched retrospective cohort study
Source: Sci Rep. 2022 Jul 7;12:11571. doi: 10.1038/s41598-022-15871-7 (PMC9262887; doi:10.1038/s41598-022-15871-7)
Supplement: Supplementary file 1 — Supplementary Information. [file 41598_2022_15871_MOESM1_ESM.docx]

| **Supplementary table S1: Baseline characteristics of propensity score-matched cohort and non-matched cohort (with blood pressure and Hemoglobin A1c included) of metformin use.** | | | | | | | |
| --- | --- | --- | --- | --- | --- | --- | --- |
|  | Non-matched cohort | | |  | Matched cohort | | |
| Metformin use | Non-user | Regular user |  |  | Non-user | Regular user |  |
| n | 3414 | 11674 |  |  | 3406 | 3406 | Standardized mean difference |
| Sex (%) |  |  |  |  |  |  | 0.031 |
| Men | 1572 (46) | 5425 (46.5) |  |  | 1570 (46.1) | 1623 (47.7) |  |
| Women | 1842 (54) | 6249 (53.5) |  |  | 1836 (53.9) | 1783 (52.3) |  |
| Mean age (standard deviation) | 68 (11.8) | 65 (10.7) |  |  | 67.9 (11.8) | 68.2 (11.7) | 0.023 |
| Biometric data (standard deviation) |  |  |  |  |  |  |  |
| Hemoglobin A1c | 6.82 (1.78) | 7.39 (2.04) |  |  | 6.83 (1.78) | 6.9 (1.75) | 0.041 |
| Systolic blood pressure | 134.73 (17.26) | 134.06 (16.72) |  |  | 134.74 (17.26) | 134.58 (16.95) | 0.009 |
| Diastolic blood pressure | 74.09 (10.21) | 75.32 (9.61) |  |  | 74.12 (10.21) | 73.95 (9.87) | 0.016 |
| Medications (%) |  |  |  |  |  |  |  |
| Insulin | 71 (2.1) | 341 (2.9) |  |  | 71 (2.1) | 70 (2.1) | 0.002 |
| NSAIDs | 420 (12.3) | 1513 (13) |  |  | 420 (12.3) | 410 (12) | 0.009 |
| Sulfonylureas | 1457 (42.7) | 6935 (59.4) |  |  | 1457 (42.8) | 1349 (39.6) | 0.064 |
| Paracetamol | 1154 (33.8) | 3649 (31.3) |  |  | 1150 (33.8) | 1136 (33.4) | 0.009 |
| Chronic conditions (%) |  |  |  |  |  |  |  |
| Renal failure | 43 (1.3) | 62 (0.5) |  |  | 40 (1.2) | 35 (1) | 0.014 |
| Heart failure | 18 (0.5) | 27 (0.2) |  |  | 17 (0.5) | 19 (0.6) | 0.008 |
| Hypertension | 2470 (72.3) | 7936 (68) |  |  | 2463 (72.3) | 2462 (72.3) | 0.001 |
| Stroke | 90 (2.6) | 160 (1.4) |  |  | 90 (2.6) | 83 (2.4) | 0.013 |
| Ischemic heart disease | 82 (2.4) | 223 (1.9) |  |  | 81 (2.4) | 78 (2.3) | 0.006 |
| Tobacco abuse | 21 (0.6) | 131 (1.1) |  |  | 21 (0.6) | 17 (0.5) | 0.016 |
| Lipid disorder | 666 (19.5) | 2210 (18.9) |  |  | 665 (19.5) | 654 (19.2) | 0.008 |
| Total knee replacement (%) | 20 (0.6) | 28 (0.2) |  |  | 20 (0.6) | 4 (0.1) |  |
| Hazard ratio of total knee replacement (95% confidence interval)^*^ | | | |  | **0.20 (0.07 – 0.58), P = 0.003** | | |
| ^*^ Derived from t-tests, chi-square tests, or Fisher's exact tests for the between group differences | | | | | | | |

| **Supplementary table S2: Baseline characteristics of propensity score-matched cohort and non-matched cohort of sulfonylurea** | | | | | | | |
| --- | --- | --- | --- | --- | --- | --- | --- |
|  | Non-matched cohort | | |  | Matched cohort | | |
| Sulfonylureas use | Non-user | Regular user |  |  | Non-user | Regular user |  |
| n | 7544 | 7987 |  |  | 6632 | 6632 | Standardized mean difference |
| Sex (%) |  |  |  |  |  |  | 0.024 |
| Men | 3276 (43.4) | 3910 (49) |  |  | 3052 (46) | 2972 (44.8) |  |
| Women | 4268 (43.4) | 4077 (49) |  |  | 3580 (46) | 3660 (44.8) |  |
| Mean age (standard deviation) | 65 (11.1) | 66.1 (11) |  |  | 65.6 (11.1) | 65.3 (11) | 0.025 |
| Biometric data (standard deviation) |  |  |  |  |  |  |  |
| Hemoglobin A1c | 7.12 (7.73) | 7.5 (2.11) |  |  | 7.09 (1.83) | 7.35 (2.16) | 0.129 |
| Systolic blood pressure | 133.29 (16.53) | 135.16 (17.04) |  |  | 133.86 (16.67) | 133.78 (16.62) | 0.005 |
| Diastolic blood pressure | 75.12 (9.67) | 75.12 (9.89) |  |  | 75.02 (9.67) | 75.09 (9.83) | 0.007 |
| Medications (%) |  |  |  |  |  |  |  |
| Insulin | 219 (2.9) | 195 (2.4) |  |  | 193 (2.9) | 178 (2.7) | 0.014 |
| NSAIDs | 967 (12.8) | 1014 (12.7) |  |  | 859 (13) | 831 (12.5) | 0.013 |
| Metformin | 5587 (74.1) | 6689 (83.7) |  |  | 5340 (80.5) | 5352 (80.7) | 0.005 |
| Paracetamol | 2436 (32.3) | 2491 (31.2) |  |  | 2146 (32.4) | 2120 (32) | 0.008 |
| Chronic conditions (%) |  |  |  |  |  |  |  |
| Renal failure | 33 (0.4) | 75 (0.9) |  |  | 32 (0.5) | 37 (0.6) | 0.010 |
| Heart failure | 23 (0.3) | 21 (0.3) |  |  | 18 (0.3) | 17 (0.3) | 0.003 |
| Hypertension | 5200 (68.9) | 5428 (68) |  |  | 4529 (68.3) | 4554 (68.7) | 0.008 |
| Stroke | 130 (1.7) | 118 (1.5) |  |  | 114 (1.7) | 104 (1.6) | 0.012 |
| Ischemic heart disease | 139 (1.8) | 168 (2.1) |  |  | 125 (1.9) | 127 (1.9) | 0.002 |
| Tobacco abuse | 73 (1) | 81 (1) |  |  | 67 (1) | 68 (1) | 0.002 |
| Lipid disorder | 1539 (20.4) | 1395 (17.5) |  |  | 1256 (18.9) | 1295 (19.5) | 0.015 |
| Total knee replacement (%) | 21 (0.3) | 24 (0.3) |  |  | 16 (0.2) | 23 (0.3) | 0.019 |
| Hazard ratio of total knee replacement (95% confidence interval) ^*^ | | | |  | **1.50 ( 0.79 – 2.85), P = 0.216** | | |
| ^*^: Adjusted for HbA1c which was unbalanced between propensity-score matched groups, i.e. standardized mean difference > 0.1 | | | | | | | |
